# Bis{μ-4,4′-dibromo-2,2′-[*o*-phenyl­enebis(nitrilo­methyl­idyne)]diphenolato}bis­[chloridomanganese(III)] *N*,*N*-dimethyl­formamide disolvate

**DOI:** 10.1107/S1600536810003247

**Published:** 2010-01-30

**Authors:** Kwang Ha

**Affiliations:** a School of Applied Chemical Engineering, The Research Institute of Catalysis, Chonnam National University, Gwangju 500-757, Republic of Korea

## Abstract

The asymmetric unit of the title compound, [Mn_2_(C_20_H_12_Br_2_N_2_O_2_)_2_Cl_2_]·2C_3_H_7_NO, contains one half of a centrosymmetric dinuclear Mn^III^ complex and an *N*,*N*-dimethyl­formamide solvent mol­ecule. In the complex, the two Mn^III^ ions are bridged by two O atoms from two symmetry-related *N*,*N*′-bis­(5-bromo­salicyl­idene)-1,2-diimino­benzene dianionic ligands with the longer Mn—O distance of 2.703 (3) Å, thus each Mn ion is six-coordinated by two N and three O atoms from the two dianionic ligands and one capping Cl atom in a distorted octa­hedral environment. The crystal structure displays inter­molecular π–π inter­actions between adjacent benzene rings, with a shortest centroid–centroid distance of 3.673 (2) Å, and inter­molecular C—H⋯O, C—H⋯ Cl and C—H⋯ Br hydrogen bonds.

## Related literature

For the crystal structure of dinuclear [Mn(salen)(H_2_O)]_2_(ClO_4_)_2_ (H_2_salen = *N*,*N*′-bis­(salicyl­idene)ethyl­enediimine), see: Shyu *et al.* (1999 ▶). For the crystal structures of 5-bromo­salicylideneimine–Mn(III) complexes, see: Dang *et al.* (2005 ▶); Hwang & Ha (2007 ▶); Mitra *et al.* (2006 ▶).
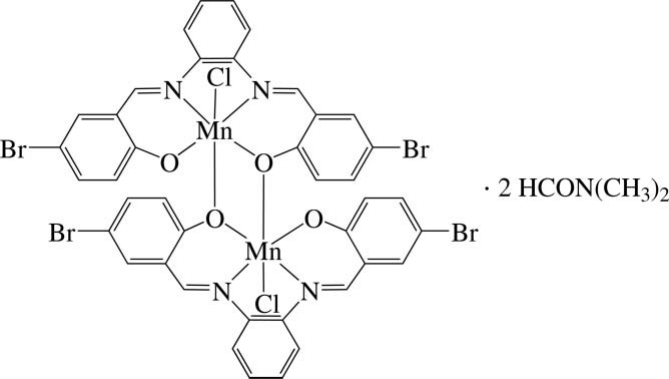


## Experimental

### 

#### Crystal data

[Mn_2_(C_20_H_12_Br_2_N_2_O_2_)_2_Cl_2_]·2C_3_H_7_NO
*M* *_r_* = 1271.24
Monoclinic, 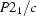

*a* = 9.7804 (6) Å
*b* = 20.1342 (12) Å
*c* = 11.8593 (6) Å
β = 90.938 (1)°
*V* = 2335.0 (2) Å^3^
*Z* = 2
Mo *K*α radiation
μ = 4.13 mm^−1^
*T* = 200 K
0.26 × 0.10 × 0.07 mm

#### Data collection

Bruker SMART 1000 CCD diffractometer
Absorption correction: multi-scan (*SADABS*; Bruker, 2000 ▶) *T*_min_ = 0.742, *T*_max_ = 1.000
17204 measured reflections
5763 independent reflections
3286 reflections with *I* > 2σ(*I*)
*R*_int_ = 0.067

#### Refinement

*R*[*F* ^2^ > 2σ(*F* ^2^)] = 0.045
*wR*(*F* ^2^) = 0.111
*S* = 1.01
5763 reflections
300 parameters
H-atom parameters constrained
Δρ_max_ = 0.73 e Å^−3^
Δρ_min_ = −0.87 e Å^−3^

### 

Data collection: *SMART* (Bruker, 2000 ▶); cell refinement: *SAINT* (Bruker, 2000 ▶); data reduction: *SAINT*; program(s) used to solve structure: *SHELXS97* (Sheldrick, 2008 ▶); program(s) used to refine structure: *SHELXL97* (Sheldrick, 2008 ▶); molecular graphics: *ORTEP-3* (Farrugia, 1997 ▶) and *PLATON* (Spek, 2009 ▶); software used to prepare material for publication: *SHELXL97*.

## Supplementary Material

Crystal structure: contains datablocks global, I. DOI: 10.1107/S1600536810003247/xu2722sup1.cif

Structure factors: contains datablocks I. DOI: 10.1107/S1600536810003247/xu2722Isup2.hkl

Additional supplementary materials:  crystallographic information; 3D view; checkCIF report

## Figures and Tables

**Table 1:** Selected bond lengths (Å)

Mn1—O1	1.869 (3)
Mn1—O2	1.884 (3)
Mn1—O2^i^	2.703 (3)
Mn1—N1	1.991 (3)
Mn1—N2	1.994 (3)
Mn1—Cl1	2.4268 (13)

Symmetry code: (i) 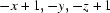.

**Table 2:** Hydrogen-bond geometry (Å, °)

*D*—H⋯*A*	*D*—H	H⋯*A*	*D*⋯*A*	*D*—H⋯*A*
C7—H7⋯Cl1^ii^	0.95	2.71	3.603 (4)	156
C12—H12⋯O3^iii^	0.95	2.54	3.468 (6)	165
C14—H14⋯O3^iii^	0.95	2.25	3.173 (5)	165
C19—H19⋯Br2^iv^	0.95	2.88	3.678 (4)	143

Symmetry codes: (ii) 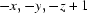; (iii) 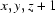; (iv) 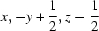.

**Fig. 1.**
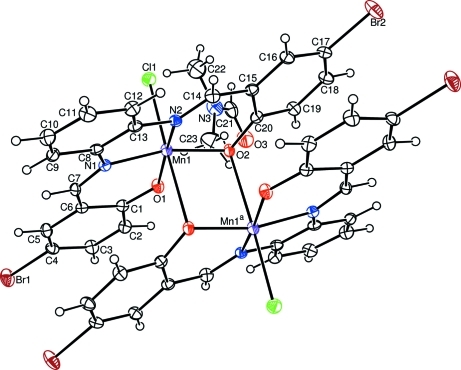
The structure of the title compound, with displacement ellipsoids drawn at the 30% probability level for non-H atoms. Unlabelled atoms and Mn1a are related to labelled atoms by the symmetry operation [symmetry code: (a) 1 - x, -y, 1 - z].

**Fig. 2.**
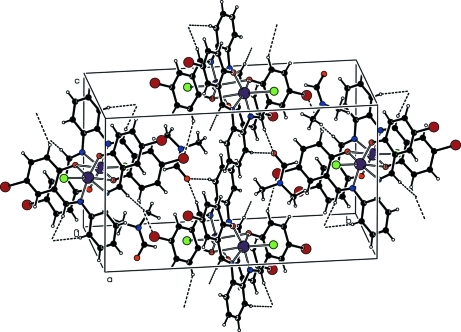
View of the unit-cell contents of the title compound. Hydrogen-bond interactions are drawn with dashed lines.

## supplementary crystallographic information

### Comment

The title compound, [Mn(C_20_H_12_Br_2_N_2_O_2_)Cl]_2_.2(C_3_H_7_NO), consists of a structurally centrosymmetric dinuclear Mn^III^ complex and two *N*,*N*-dimethylformamide solvent molecules, and the asymmetric unit contains one half of the formula unit (Fig. 1). In the complex, two Mn^III^ ions are bridged by two phenolic O atoms from two symmetry-related *N*,*N*'-bis(5-bromosalicylidene)-1,2-diiminobenzene dianionic ligands, and the distance between the Mn atoms is 3.5277 (10) Å. Each Mn ion is six-coordinated by two N and three O atoms from the two dianionic ligands and one capping Cl atom in a distorted octahedral environment (Table 1). While the two Mn—N bond distances are nearly equal [1.991 (3) Å and 1.994 (3) Å], the three Mn—O bond lengths are somewhat different. The equatorial Mn1—O1/O2 bonds [1.869 (3) Å and 1.884 (3) Å] are considerably shorter than the axial Mn1—O2^a^ bond [Symmetry code: (a) 1 - x, -y, 1 - z; 2.703 (3) Å]. Within the equatorial plane, the chelating angles lie in the range of 82.42 (14)°–92.38 (14)° and the <O1—Mn1—O2 bond angle is 90.84 (12)°. The apical <Cl1—Mn1—O2^a^ bond angle is 173.85 (7)°. The crystal structure displays intermolecular π-π interactions between adjacent benzene rings, with a shortest centroid-centroid distance of 3.673 (2) Å, and intermolecular C—H···O/Cl/Br hydrogen bonds (Fig. 2 and Table 2).

### Experimental

Mn(CH_3_CO_2_)_3_.2H_2_O (0.50 g, 1.86 mmol), NaCl (0.11 g, 1.88 mmol) and *N*,*N*'-bis(5-bromosalicylidene)-1,2-diiminobenzene (0.89 g, 1.88 mmol) in EtOH (50 ml) and acetone (20 ml) were stirred for 2 h at room temperature. The formed precipitate was separated by filtration and washed with acetone and ether and dried under vacuum, to give a dark brown powder (1.02 g). Crystals suitable for X-ray analysis were obtained by slow evaporation under vacuum from an *N*,*N*-dimethylformamide solution.

### Refinement

H atoms were positioned geometrically and allowed to ride on their respective parent atoms [C—H = 0.95 Å (CH) or 0.98 Å (CH_3_) and U_iso_(H) = 1.2U_eq_(CH) or 1.5U_eq_(CH_3_)].

### Crystal data

**Table:**

[Mn_2_(C_20_H_12_Br_2_N_2_O_2_)_2_Cl_2_]·2C_3_H_7_NO	*F*(000) = 1256
*M**_r_* = 1271.24	*D*_x_ = 1.808 Mg m^−^^3^
Monoclinic, *P*2_1_/*c*	Mo *K*α radiation, λ = 0.71073 Å
Hall symbol: -P 2ybc	Cell parameters from 3984 reflections
*a* = 9.7804 (6) Å	θ = 2.3–27.8°
*b* = 20.1342 (12) Å	µ = 4.13 mm^−^^1^
*c* = 11.8593 (6) Å	*T* = 200 K
β = 90.938 (1)°	Block, brown
*V* = 2335.0 (2) Å^3^	0.26 × 0.10 × 0.07 mm
*Z* = 2	

### Data collection

**Table:**

Bruker SMART 1000 CCD diffractometer	5763 independent reflections
Radiation source: fine-focus sealed tube	3286 reflections with *I* > 2σ(*I*)
graphite	*R*_int_ = 0.067
φ and ω scans	θ_max_ = 28.3°, θ_min_ = 2.0°
Absorption correction: multi-scan (*SADABS*; Bruker, 2000)	*h* = −13→13
*T*_min_ = 0.742, *T*_max_ = 1.000	*k* = −26→25
17204 measured reflections	*l* = −9→15

### Refinement

**Table:**

Refinement on *F*^2^	Primary atom site location: structure-invariant direct methods
Least-squares matrix: full	Secondary atom site location: difference Fourier map
*R*[*F*^2^ > 2σ(*F*^2^)] = 0.045	Hydrogen site location: inferred from neighbouring sites
*wR*(*F*^2^) = 0.111	H-atom parameters constrained
*S* = 1.01	*w* = 1/[σ^2^(*F*_o_^2^) + (0.0372*P*)^2^] where *P* = (*F*_o_^2^ + 2*F*_c_^2^)/3
5763 reflections	(Δ/σ)_max_ < 0.001
300 parameters	Δρ_max_ = 0.73 e Å^−^^3^
0 restraints	Δρ_min_ = −0.87 e Å^−^^3^

### Special details

**Table:**

Geometry. All esds (except the esd in the dihedral angle between two l.s. planes) are estimated using the full covariance matrix. The cell esds are taken into account individually in the estimation of esds in distances, angles and torsion angles; correlations between esds in cell parameters are only used when they are defined by crystal symmetry. An approximate (isotropic) treatment of cell esds is used for estimating esds involving l.s. planes.
Refinement. Refinement of *F*^2^ against ALL reflections. The weighted *R*-factor wR and goodness of fit *S* are based on *F*^2^, conventional *R*-factors *R* are based on *F*, with *F* set to zero for negative *F*^2^. The threshold expression of *F*^2^ > σ(*F*^2^) is used only for calculating *R*-factors(gt) etc. and is not relevant to the choice of reflections for refinement. *R*-factors based on *F*^2^ are statistically about twice as large as those based on *F*, and *R*- factors based on ALL data will be even larger.

### Fractional atomic coordinates and isotropic or equivalent isotropic displacement parameters (Å^2^)

**Table:**

	*x*	*y*	*z*	*U*_iso_*/*U*_eq_	
Mn1	0.33723 (7)	0.03642 (3)	0.48099 (5)	0.02156 (17)	
Br1	−0.07312 (5)	−0.20087 (3)	0.13904 (4)	0.04294 (17)	
Br2	0.80307 (6)	0.30297 (3)	0.65558 (4)	0.04307 (17)	
Cl1	0.20464 (11)	0.13541 (6)	0.43825 (9)	0.0301 (3)	
O1	0.3251 (3)	−0.00461 (15)	0.3398 (2)	0.0252 (7)	
O2	0.5128 (3)	0.06888 (14)	0.4485 (2)	0.0225 (7)	
N1	0.1887 (3)	−0.01986 (17)	0.5427 (3)	0.0200 (8)	
N2	0.3604 (3)	0.06227 (17)	0.6425 (3)	0.0200 (8)	
C1	0.2337 (4)	−0.0470 (2)	0.3001 (3)	0.0212 (10)	
C2	0.2434 (5)	−0.0675 (2)	0.1874 (3)	0.0259 (11)	
H2	0.3143	−0.0498	0.1425	0.031*	
C3	0.1542 (5)	−0.1118 (2)	0.1405 (4)	0.0276 (11)	
H3	0.1631	−0.1250	0.0641	0.033*	
C4	0.0485 (4)	−0.1379 (2)	0.2063 (4)	0.0253 (10)	
C5	0.0369 (4)	−0.1213 (2)	0.3164 (4)	0.0262 (11)	
H5	−0.0331	−0.1407	0.3602	0.031*	
C6	0.1289 (4)	−0.0750 (2)	0.3666 (4)	0.0227 (10)	
C7	0.1164 (4)	−0.0620 (2)	0.4843 (3)	0.0221 (10)	
H7	0.0489	−0.0863	0.5235	0.027*	
C8	0.1754 (4)	−0.0142 (2)	0.6626 (3)	0.0203 (10)	
C9	0.0778 (4)	−0.0469 (2)	0.7259 (4)	0.0243 (10)	
H9	0.0126	−0.0751	0.6902	0.029*	
C10	0.0767 (5)	−0.0378 (2)	0.8416 (4)	0.0294 (11)	
H10	0.0101	−0.0600	0.8854	0.035*	
C11	0.1716 (5)	0.0031 (2)	0.8938 (4)	0.0294 (11)	
H11	0.1699	0.0085	0.9733	0.035*	
C12	0.2684 (5)	0.0360 (2)	0.8326 (4)	0.0267 (11)	
H12	0.3339	0.0636	0.8694	0.032*	
C13	0.2693 (4)	0.0283 (2)	0.7156 (3)	0.0211 (10)	
C14	0.4430 (4)	0.1081 (2)	0.6771 (3)	0.0241 (10)	
H14	0.4422	0.1191	0.7550	0.029*	
C15	0.5364 (4)	0.1440 (2)	0.6067 (3)	0.0223 (10)	
C16	0.6084 (4)	0.1977 (2)	0.6544 (4)	0.0253 (10)	
H16	0.5926	0.2104	0.7302	0.030*	
C17	0.7015 (5)	0.2317 (2)	0.5916 (4)	0.0246 (10)	
C18	0.7236 (5)	0.2148 (2)	0.4793 (4)	0.0272 (11)	
H18	0.7852	0.2401	0.4354	0.033*	
C19	0.6564 (4)	0.1618 (2)	0.4328 (4)	0.0250 (10)	
H19	0.6724	0.1504	0.3564	0.030*	
C20	0.5642 (4)	0.1237 (2)	0.4949 (3)	0.0218 (10)	
O3	0.4905 (4)	0.1586 (2)	−0.0718 (3)	0.0526 (11)	
N3	0.3774 (5)	0.1726 (2)	0.0910 (3)	0.0434 (11)	
C21	0.4185 (5)	0.1909 (3)	−0.0094 (4)	0.0394 (13)	
H21	0.3893	0.2332	−0.0358	0.047*	
C22	0.2859 (6)	0.2150 (3)	0.1564 (5)	0.0637 (19)	
H22A	0.2676	0.2561	0.1148	0.096*	
H22B	0.3294	0.2255	0.2293	0.096*	
H22C	0.1997	0.1915	0.1689	0.096*	
C23	0.4163 (8)	0.1103 (3)	0.1379 (5)	0.078 (2)	
H23A	0.4858	0.0897	0.0906	0.117*	
H23B	0.3361	0.0813	0.1413	0.117*	
H23C	0.4539	0.1170	0.2142	0.117*	

### Atomic displacement parameters (Å^2^)

**Table:**

	*U*^11^	*U*^22^	*U*^33^	*U*^12^	*U*^13^	*U*^23^
Mn1	0.0221 (4)	0.0250 (4)	0.0177 (4)	−0.0037 (3)	0.0021 (3)	−0.0023 (3)
Br1	0.0400 (3)	0.0505 (4)	0.0382 (3)	−0.0164 (3)	−0.0034 (2)	−0.0141 (3)
Br2	0.0603 (4)	0.0382 (3)	0.0309 (3)	−0.0249 (3)	0.0080 (2)	−0.0088 (2)
Cl1	0.0297 (6)	0.0288 (7)	0.0318 (7)	0.0011 (5)	0.0009 (5)	0.0034 (5)
O1	0.0263 (17)	0.0306 (19)	0.0187 (17)	−0.0060 (14)	−0.0014 (13)	−0.0034 (13)
O2	0.0200 (16)	0.0275 (18)	0.0201 (16)	−0.0051 (13)	0.0049 (12)	−0.0089 (13)
N1	0.0218 (19)	0.020 (2)	0.0180 (19)	0.0009 (16)	0.0016 (15)	0.0020 (15)
N2	0.0219 (19)	0.018 (2)	0.020 (2)	0.0006 (16)	0.0005 (15)	0.0017 (15)
C1	0.023 (2)	0.023 (3)	0.017 (2)	0.0026 (19)	−0.0062 (18)	0.0007 (18)
C2	0.027 (3)	0.032 (3)	0.019 (2)	0.000 (2)	0.0018 (19)	−0.0005 (19)
C3	0.034 (3)	0.034 (3)	0.015 (2)	−0.003 (2)	−0.003 (2)	−0.0078 (19)
C4	0.029 (3)	0.022 (3)	0.025 (3)	−0.001 (2)	−0.007 (2)	−0.0041 (19)
C5	0.023 (2)	0.029 (3)	0.026 (3)	−0.004 (2)	−0.0028 (19)	0.000 (2)
C6	0.023 (2)	0.019 (3)	0.026 (3)	0.0016 (19)	0.0013 (19)	−0.0022 (18)
C7	0.020 (2)	0.022 (3)	0.024 (3)	−0.0032 (19)	0.0052 (18)	0.0011 (18)
C8	0.025 (2)	0.019 (2)	0.017 (2)	0.0045 (19)	0.0041 (18)	0.0002 (17)
C9	0.021 (2)	0.024 (3)	0.029 (3)	0.0021 (19)	0.0034 (19)	0.0030 (19)
C10	0.034 (3)	0.033 (3)	0.021 (3)	0.000 (2)	0.010 (2)	0.006 (2)
C11	0.039 (3)	0.029 (3)	0.019 (2)	−0.001 (2)	0.005 (2)	0.000 (2)
C12	0.029 (3)	0.031 (3)	0.020 (2)	0.002 (2)	0.0002 (19)	−0.002 (2)
C13	0.026 (2)	0.020 (3)	0.018 (2)	0.0038 (19)	0.0045 (18)	0.0005 (18)
C14	0.031 (3)	0.030 (3)	0.012 (2)	0.000 (2)	−0.0021 (18)	−0.0038 (18)
C15	0.025 (2)	0.021 (3)	0.021 (2)	0.0032 (19)	0.0020 (18)	0.0026 (18)
C16	0.031 (3)	0.025 (3)	0.020 (2)	−0.001 (2)	0.0001 (19)	−0.0046 (19)
C17	0.032 (3)	0.018 (2)	0.024 (3)	0.000 (2)	0.001 (2)	−0.0014 (19)
C18	0.037 (3)	0.021 (3)	0.023 (3)	−0.004 (2)	0.006 (2)	0.0027 (19)
C19	0.031 (3)	0.028 (3)	0.017 (2)	−0.003 (2)	0.0021 (19)	0.0000 (19)
C20	0.022 (2)	0.024 (3)	0.020 (2)	0.002 (2)	−0.0001 (18)	0.0000 (18)
O3	0.059 (3)	0.065 (3)	0.033 (2)	−0.010 (2)	0.0096 (19)	−0.0239 (19)
N3	0.057 (3)	0.046 (3)	0.027 (2)	−0.011 (2)	0.010 (2)	−0.001 (2)
C21	0.043 (3)	0.047 (4)	0.028 (3)	−0.014 (3)	−0.005 (2)	−0.004 (2)
C22	0.073 (5)	0.069 (5)	0.050 (4)	−0.009 (4)	0.026 (3)	−0.006 (3)
C23	0.117 (7)	0.058 (5)	0.059 (5)	−0.005 (4)	−0.006 (4)	0.014 (4)

### Geometric parameters (Å, °)

**Table:**

Mn1—O1	1.869 (3)	C10—C11	1.380 (6)
Mn1—O2	1.884 (3)	C10—H10	0.9500
Mn1—O2^i^	2.703 (3)	C11—C12	1.373 (6)
Mn1—N1	1.991 (3)	C11—H11	0.9500
Mn1—N2	1.994 (3)	C12—C13	1.397 (6)
Mn1—Cl1	2.4268 (13)	C12—H12	0.9500
Br1—C4	1.904 (4)	C14—C15	1.441 (6)
Br2—C17	1.896 (4)	C14—H14	0.9500
O1—C1	1.318 (5)	C15—C16	1.406 (6)
O2—C20	1.328 (5)	C15—C20	1.418 (6)
N1—C7	1.297 (5)	C16—C17	1.369 (6)
N1—C8	1.435 (5)	C16—H16	0.9500
N2—C14	1.290 (5)	C17—C18	1.395 (6)
N2—C13	1.429 (5)	C18—C19	1.366 (6)
C1—C2	1.403 (5)	C18—H18	0.9500
C1—C6	1.420 (6)	C19—C20	1.402 (6)
C2—C3	1.361 (6)	C19—H19	0.9500
C2—H2	0.9500	O3—C21	1.217 (6)
C3—C4	1.406 (6)	N3—C21	1.316 (6)
C3—H3	0.9500	N3—C23	1.422 (7)
C4—C5	1.355 (6)	N3—C22	1.466 (7)
C5—C6	1.419 (6)	C21—H21	0.9500
C5—H5	0.9500	C22—H22A	0.9800
C6—C7	1.428 (5)	C22—H22B	0.9800
C7—H7	0.9500	C22—H22C	0.9800
C8—C9	1.389 (6)	C23—H23A	0.9800
C8—C13	1.397 (6)	C23—H23B	0.9800
C9—C10	1.384 (6)	C23—H23C	0.9800
C9—H9	0.9500		
			
O1—Mn1—O2	90.84 (12)	C11—C10—H10	119.8
O1—Mn1—N1	92.38 (14)	C9—C10—H10	119.8
O2—Mn1—N1	161.07 (14)	C12—C11—C10	121.0 (4)
O1—Mn1—N2	168.61 (14)	C12—C11—H11	119.5
O2—Mn1—N2	90.93 (13)	C10—C11—H11	119.5
N1—Mn1—N2	82.42 (14)	C11—C12—C13	119.2 (4)
O1—Mn1—Cl1	98.63 (10)	C11—C12—H12	120.4
O2—Mn1—Cl1	99.10 (10)	C13—C12—H12	120.4
N1—Mn1—Cl1	98.84 (10)	C12—C13—C8	120.0 (4)
N2—Mn1—Cl1	92.20 (10)	C12—C13—N2	124.3 (4)
O1—Mn1—O2^i^	87.52 (11)	C8—C13—N2	115.6 (4)
O2—Mn1—O2^i^	81.01 (12)	N2—C14—C15	125.0 (4)
N1—Mn1—O2^i^	80.51 (11)	N2—C14—H14	117.5
N2—Mn1—O2^i^	81.65 (11)	C15—C14—H14	117.5
Cl1—Mn1—O2^i^	173.85 (7)	C16—C15—C20	119.7 (4)
C1—O1—Mn1	129.7 (3)	C16—C15—C14	118.1 (4)
C20—O2—Mn1	122.9 (3)	C20—C15—C14	122.1 (4)
C7—N1—C8	121.5 (3)	C17—C16—C15	120.0 (4)
C7—N1—Mn1	124.8 (3)	C17—C16—H16	120.0
C8—N1—Mn1	113.5 (3)	C15—C16—H16	120.0
C14—N2—C13	122.9 (4)	C16—C17—C18	120.8 (4)
C14—N2—Mn1	123.6 (3)	C16—C17—Br2	120.6 (3)
C13—N2—Mn1	113.3 (3)	C18—C17—Br2	118.6 (3)
O1—C1—C2	118.3 (4)	C19—C18—C17	119.7 (4)
O1—C1—C6	123.4 (4)	C19—C18—H18	120.2
C2—C1—C6	118.2 (4)	C17—C18—H18	120.2
C3—C2—C1	121.9 (4)	C18—C19—C20	121.7 (4)
C3—C2—H2	119.0	C18—C19—H19	119.1
C1—C2—H2	119.0	C20—C19—H19	119.1
C2—C3—C4	119.4 (4)	O2—C20—C19	118.6 (4)
C2—C3—H3	120.3	O2—C20—C15	123.4 (4)
C4—C3—H3	120.3	C19—C20—C15	117.9 (4)
C5—C4—C3	121.1 (4)	C21—N3—C23	121.1 (5)
C5—C4—Br1	120.4 (3)	C21—N3—C22	121.0 (5)
C3—C4—Br1	118.4 (3)	C23—N3—C22	117.9 (5)
C4—C5—C6	120.3 (4)	O3—C21—N3	126.1 (6)
C4—C5—H5	119.9	O3—C21—H21	116.9
C6—C5—H5	119.9	N3—C21—H21	116.9
C5—C6—C1	119.1 (4)	N3—C22—H22A	109.5
C5—C6—C7	117.9 (4)	N3—C22—H22B	109.5
C1—C6—C7	122.9 (4)	H22A—C22—H22B	109.5
N1—C7—C6	126.0 (4)	N3—C22—H22C	109.5
N1—C7—H7	117.0	H22A—C22—H22C	109.5
C6—C7—H7	117.0	H22B—C22—H22C	109.5
C9—C8—C13	119.9 (4)	N3—C23—H23A	109.5
C9—C8—N1	125.0 (4)	N3—C23—H23B	109.5
C13—C8—N1	115.1 (4)	H23A—C23—H23B	109.5
C10—C9—C8	119.4 (4)	N3—C23—H23C	109.5
C10—C9—H9	120.3	H23A—C23—H23C	109.5
C8—C9—H9	120.3	H23B—C23—H23C	109.5
C11—C10—C9	120.4 (4)		
			
O2—Mn1—O1—C1	170.3 (4)	C8—N1—C7—C6	−175.0 (4)
N1—Mn1—O1—C1	8.9 (4)	Mn1—N1—C7—C6	−0.6 (6)
N2—Mn1—O1—C1	71.4 (8)	C5—C6—C7—N1	−177.7 (4)
Cl1—Mn1—O1—C1	−90.4 (3)	C1—C6—C7—N1	6.2 (7)
O2^i^—Mn1—O1—C1	89.3 (4)	C7—N1—C8—C9	−6.7 (6)
O1—Mn1—O2—C20	153.2 (3)	Mn1—N1—C8—C9	178.3 (3)
N1—Mn1—O2—C20	−107.0 (5)	C7—N1—C8—C13	173.8 (4)
N2—Mn1—O2—C20	−38.1 (3)	Mn1—N1—C8—C13	−1.1 (4)
Cl1—Mn1—O2—C20	54.3 (3)	C13—C8—C9—C10	−1.3 (6)
O2^i^—Mn1—O2—C20	−119.5 (3)	N1—C8—C9—C10	179.3 (4)
O1—Mn1—N1—C7	−5.4 (4)	C8—C9—C10—C11	−0.1 (7)
O2—Mn1—N1—C7	−105.0 (5)	C9—C10—C11—C12	0.4 (7)
N2—Mn1—N1—C7	−175.2 (4)	C10—C11—C12—C13	0.7 (7)
Cl1—Mn1—N1—C7	93.7 (3)	C11—C12—C13—C8	−2.1 (6)
O2^i^—Mn1—N1—C7	−92.5 (4)	C11—C12—C13—N2	176.9 (4)
O1—Mn1—N1—C8	169.4 (3)	C9—C8—C13—C12	2.4 (6)
O2—Mn1—N1—C8	69.8 (5)	N1—C8—C13—C12	−178.1 (4)
N2—Mn1—N1—C8	−0.5 (3)	C9—C8—C13—N2	−176.7 (4)
Cl1—Mn1—N1—C8	−91.5 (3)	N1—C8—C13—N2	2.8 (5)
O2^i^—Mn1—N1—C8	82.3 (3)	C14—N2—C13—C12	−6.5 (7)
O1—Mn1—N2—C14	122.9 (7)	Mn1—N2—C13—C12	177.8 (3)
O2—Mn1—N2—C14	24.0 (4)	C14—N2—C13—C8	172.6 (4)
N1—Mn1—N2—C14	−173.8 (4)	Mn1—N2—C13—C8	−3.2 (5)
Cl1—Mn1—N2—C14	−75.2 (4)	C13—N2—C14—C15	−179.8 (4)
O2^i^—Mn1—N2—C14	104.8 (4)	Mn1—N2—C14—C15	−4.4 (6)
O1—Mn1—N2—C13	−61.4 (8)	N2—C14—C15—C16	172.8 (4)
O2—Mn1—N2—C13	−160.3 (3)	N2—C14—C15—C20	−11.8 (7)
N1—Mn1—N2—C13	1.9 (3)	C20—C15—C16—C17	2.4 (7)
Cl1—Mn1—N2—C13	100.6 (3)	C14—C15—C16—C17	177.8 (4)
O2^i^—Mn1—N2—C13	−79.5 (3)	C15—C16—C17—C18	1.6 (7)
Mn1—O1—C1—C2	176.1 (3)	C15—C16—C17—Br2	−178.4 (3)
Mn1—O1—C1—C6	−6.3 (6)	C16—C17—C18—C19	−3.0 (7)
O1—C1—C2—C3	179.1 (4)	Br2—C17—C18—C19	177.0 (4)
C6—C1—C2—C3	1.4 (7)	C17—C18—C19—C20	0.4 (7)
C1—C2—C3—C4	0.3 (7)	Mn1—O2—C20—C19	−149.3 (3)
C2—C3—C4—C5	−2.2 (7)	Mn1—O2—C20—C15	34.1 (5)
C2—C3—C4—Br1	−179.1 (3)	C18—C19—C20—O2	−173.3 (4)
C3—C4—C5—C6	2.3 (7)	C18—C19—C20—C15	3.5 (7)
Br1—C4—C5—C6	179.2 (3)	C16—C15—C20—O2	171.8 (4)
C4—C5—C6—C1	−0.6 (6)	C14—C15—C20—O2	−3.5 (7)
C4—C5—C6—C7	−176.8 (4)	C16—C15—C20—C19	−4.8 (6)
O1—C1—C6—C5	−178.8 (4)	C14—C15—C20—C19	179.9 (4)
C2—C1—C6—C5	−1.2 (6)	C23—N3—C21—O3	0.3 (9)
O1—C1—C6—C7	−2.8 (7)	C22—N3—C21—O3	−177.5 (5)
C2—C1—C6—C7	174.8 (4)		

Symmetry codes: (i) −*x*+1, −*y*, −*z*+1.

### Hydrogen-bond geometry (Å, °)

**Table:**

*D*—H···*A*	*D*—H	H···*A*	*D*···*A*	*D*—H···*A*
C7—H7···Cl1^ii^	0.95	2.71	3.603 (4)	156
C12—H12···O3^iii^	0.95	2.54	3.468 (6)	165
C14—H14···O3^iii^	0.95	2.25	3.173 (5)	165
C19—H19···Br2^iv^	0.95	2.88	3.678 (4)	143

Symmetry codes: (ii) −*x*, −*y*, −*z*+1; (iii) *x*, *y*, *z*+1; (iv) *x*, −*y*+1/2, *z*−1/2.
